# Multimodality Imaging Approach in Evaluation of Post-Traumatic Bronchobiliary Fistulas

**DOI:** 10.7759/cureus.10168

**Published:** 2020-08-31

**Authors:** Savas Ozdemir, Ashley Way, Dheeraj Gopireddy

**Affiliations:** 1 Radiology, University of Florida College of Medicine, Jacksonville, USA

**Keywords:** post-traumatic bronchobiliary fistula, hepatobiliary scintigraphy, spect/ct, computed tomography, magnetic resonance imaging

## Abstract

Post-traumatic bronchobiliary fistulas (BBF) are extremely rare with high morbidity and mortality rates. Accurate and timely diagnosis of these entities is critical for appropriate treatment, which usually requires a multidisciplinary approach. We describe two post-traumatic cases using a multimodality approach including computed tomography (CT), magnetic resonance imaging (MRI)/Magnetic Resonance Cholangiopancreatography (MRCP), and hepatobiliary scintigraphy with specific emphasis on the imaging features for each modality. Management of hepatobiliary fistulas is complex, involving extensive diagnostic work up followed by a conservative and/or surgical approach.

## Introduction

Bronchobiliary fistula (BBF) is communication of biliary system and bronchial tree. It was first described by Peacock in 1850 [1]. Common causes of acquired BBF are hepatic neoplasms, bile duct obstruction, cholangiolithiasis, hepatic hydatidosis, trauma and chronic pancreatitis [2]. Post-traumatic BBFs are extremely rare. Computed tomography (CT) is used for initial evaluation of liver and biliary system in patients with history of penetrating trauma, providing critical anatomical information on the pattern of hepatic and biliary injuries. Although magnetic resonance imaging (MRI)/magnetic resonance cholangiopancreatography (MRCP) is not the first line of imaging in the acute trauma setting, it is utilized acutely when initial CT demonstrate signs of bile leak [3]. Hepatobiliary scintigraphy is very useful in evaluation of suspected BBFs [4-6]. One of the advantages of hepatobiliary scintigraphy is ability to obtain sequential dynamic functional images for a prolonged time. Bile movement can be visualized in real time to confirm the communication between biliary system and bronchial tree. Additional imaging with single positron emission computed tomography (SPECT/CT) may reveal exact location of fistulous communications, therefore confirming the diagnosis with high diagnostic accuracy [5].

## Case presentation

Case 1

**Figure 1:**
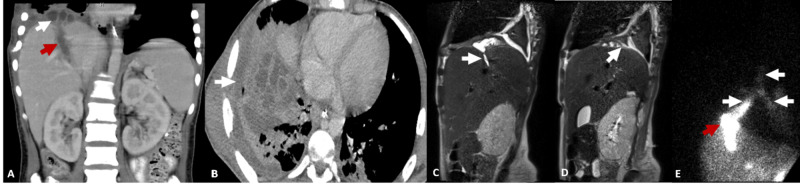
A 16-year-old male with history of penetrating injury (gunshot) to the right upper quadrant and right lower hemithorax complicated by biliary leak, diaphragmatic injury, and bilomas. (A) Coronal contrast-enhanced CT shows a fluid filled track (red arrow) and subdiaphragmatic multiloculated fluid collection (white arrow). (B) Axial contrast-enhanced CT shows a subdiaphragmatic multiloculated fluid collection (white arrow). (C) Sagittal Single Shot Fast Spin ECHO (SSFSE) images show a biliary tract extending to subdiapragmatic fluid collection (white arrow) and (D) communication between the subhepatic fluid collection and pleural cavity (white arrow). (E) Anterior planar image of hepatobiliary scintigraphy shows a focal area of increased activity in the subdiaphragmatic region, consistent with biloma (red) and abnormal activity in the bronchial tree, consistent with bronchobiliary fistula (white arrows).

A 16-year-old male with history of penetrating injury (gunshot) to the right upper quadrant and right lower hemithorax complicated by biliary leak, diaphragmatic injury, and bilomas. Initial contrast-enhanced CT demonstrated multiloculated fluid collections in the right subphrenic space and small pleural effusion (Figure 1A, 1B ). This finding was also further confirmed with abdominal MRI and MRCP, which demonstrated a direct communication between the anterior division of the right hepatic duct and right pleural space (Figure 1C, 1D ). These collections were drained percutaneously and fluid analysis demonstrated infected bilomas. However, the patient developed bilioptysis which was confirmed with bronchoscopy and fluid analysis. Subsequently, a hepatobiliary scintigraphy was positive for BBF which confirmed abnormal communication at the level of the distal right lower lobe bronchus (Figure 1E). The patient was managed with multiple biliary drains and stents to aid in decompression of the biliary system with subsequent resolution of the bronchobiliary fistula.

Case 2

A 40-year-old male and victim of gun violence with surgical history of laparotomy, left hepatectomy, cholecystectomy and removal of two foreign bodies. Six days later, he underwent an endoscopic retrograde cholangiopancreatography (ERCP) which revealed extravasation of contrast from the common bile duct and he returned to the operating room for drainage of intraabdominal abscesses and a Roux-en-Y hepaticojejunostomy.

Since then over several months he suffered from many hepatic abscesses, bilomas, cholangitis, and gram negative bacteremia, primarily managed with percutaneous drains and antibiotics. His clinical course was further complicated by misplaced drains and a biliarycutaneous fistula.

**Figure 2:**
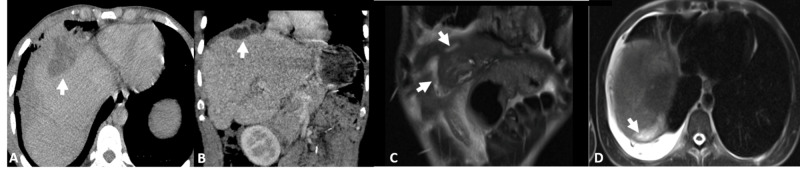
A 40-year-old male and victim of gun violence with surgical history of laparotomy, left hepatectomy, cholecystectomy and removal of two foreign bodies. (A) Axial and (B) coronal contrast-enhanced CT show a subdiaphragmatic multiloculated fluid collection (white arrows). (C) Coronal Single Shot Fast Spin ECHO (SSFSE) image shows biliary tract extending to subdiaphragmatic fluid collection (white arrows). (D)  Axial SSFSE image shows communication between the subhepatic fluid collection and pleural cavity (white arrow).

**Figure 3:**
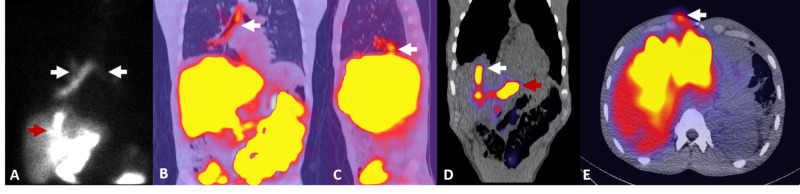
A 40-year-old male and victim of gun violence with surgical history of laparotomy, left hepatectomy, cholecystectomy and removal of two foreign bodies. (A) Anterior planar image of hepatobiliary scintigraphy shows abnormal activity which partially fills subdiaphragmatic fluid collection (red arrow) and abnormal activity in the bronchial tree, consistent with bronchobiliary fistula (white arrows). (B) Coronal SPECT/CT shows activity in the bronchial tree (white arrow). (C) Sagittal SPECT/CT reveals location of bronchobiliary fistula (white arrow). (D) Coronal SPECT/CT shows subhepatic biliary fluid collection (red arrow) and extention of biliary tract to hepatic dome (white arrow). (E) Axial SPECT/CT reveals biliarycutaneus fistula (white arrow).

Multiple admissions were made for management of the biliarycutaneous fistula, a most recent CT of the abdomen and pelvis demonstrated loculated collection in the hepatic dome extending into the right subdiaphragmatic space (Figure 2A, 2B). This was also confirmed on MRI of the abdomen (Figure 2C, 2D). His course was further managed with a percutaneous CT guided drainage which revealed an infected biloma. A hepatobiliary scintigraphy (Figure 3A) and SPECT/CT (Figure 3B, 3C, 3D, 3E) confirmed progressive accumulation of activity extending into the right subhepatic space, right hemithorax/bronchial system and anterior abdominal wall in the epigastric region, consistent with bile leak to the subhepatic region, bronchobiliary fistula and biliarycutaneous fistula. Patient was found to have pseudomonas in culture. He was discharged to complete extended course of antibiotic treatment by home infusion. 

## Discussion

There is paucity of large case series in literature since post-traumatic BBFs are extremely uncommon. Most of the publications consists of case reports and small series. A systematic literature review of 68 cases published in 30 years by Liao et al. revealed only seven cases (10.2%) secondary to trauma [2].

A significant clinical symptoms in patients with BBFs is bilioptysis. Bilioptysis is presence of bile in the sputum and believed to be pathognomonic finding for BBFs. Other common symptoms include irritating cough, fever, and jaundice [2].

Penetrating injuries rather than blunt traumas are reported in etiology of bronchopulmonary fistulas [7]. Both of our cases are result of penetrating gunshot wounds to the right upper quadrant.

Initial evaluation usually starts with CT which is useful to demonstrate liver injury, abdominal and thoracic fluid collections, as well as abnormal lung findings. The widespread availability, decreased variability between operators, imaging speed, and relatively few contraindications make it ideal in immediate traumatic assessment [3,8]. However, it rarely depicts BBF directly [4]. Instead, CT reveals several indirect findings characteristic of a biliary injury which may include perihepatic or intrahepatic fluid collections and ascites in acute phase, progressive growth of perihepatic or intraparenchymal fluid collection on follow-up imaging, and/or other secondary signs of biliary injury [3]. Diaphragmatic disruption caused by laceration associated with right pleural effusion must raise the possibility of biliarypleural fistula [9]. Additionally, CT is also helpful in management of these complex post-traumatic collections for placement of external drains.

Conventional MRI of the abdomen with MRCP (Magnetic Resonance Cholangiopancreatography) can provide good anatomical information of the biliary system. However, contrast-enhanced magnetic resonance cholangiography using hepatobiliary contrast agents was reported to demonstrate bronchobiliary fistula since it depicts biliary excretion from injured ducts [10]. Unfortunately, limitations of MRI still exist including weak/variable filing of bile ducts secondary to reduced hepatic function, potential interference with T2-weighted images secondary to T2 shortening effect, as well as general contraindications to MRI including certain implanted cardiac devises or bullet fragments in the setting of trauma [11].

Hepatobiliary scintigraphy, similar to MR cholangiopancreatography with hepatobiliary contrast agents provide functional information. Image acquisition may be obtained for a prolonged time to trace bile movement. The precise location of bronchobiliary connection could not be confidently determined with this method even when SPECT was performed in the past. SPECT/CT as a hybrid imaging technique combining the functional information of hepatobiliary scintigraphy and anatomical information of CT overcomes the shortcoming and may clearly define fistulous tract [5].

Management of BBF varies from a conservative approach to definitive surgical management. The conservative approach is preferred in the initial management of bile leaks. Biliary decompression or diversion using endoscopic sphincterotomy, biliary stent placement, or nasobiliary drainage and percutaneous image-guided catheter collections alone may be successful. More definitive treatment would require surgical resection of the fistula with pulmonary segmentectomy, especially in the presence of lung injury [12-15]. Recently, more advanced and less invasive approaches are being tried including transhepatic embolization of the fistulas [16].

## Conclusions

A multimodality approach is critical for stepwise diagnosis of BBFs. Although CT is used in initial evaluation of BBFs, hepatobiliary scintigraphy can confirm biliary leaks and differentiate bilomas from other fluid collections. Bile excretion and movement may be traced by sequential dynamic imaging. SPECT and more recently SPECT/CT may reveal connections between the bile ducts, bilomas and other structures such as bronchial tree and may play an important role in management of complex cases. MR cholangiopancreatography with hepatobiliary contrast agents also allows dynamic biliary imaging. It is an alternative to hepatobiliary scintigraphy and SPECT/CT and may demonstrate fistulous communications of the biliary system with high accuracy.
